# Ex vivo MR microscopy of a human brain with multiple sclerosis: Visualizing individual cells in tissue using intrinsic iron

**DOI:** 10.1016/j.neuroimage.2020.117285

**Published:** 2020-08-20

**Authors:** Govind Nair, Stephen Dodd, Seung-Kwon Ha, Alan P Koretsky, Daniel S Reich

**Affiliations:** a Quantitative MRI Core Facility, National Institute of Neurological Disorders and Stroke, National Institutes of Health, Bethesda, MD, 20892, United States; b Laboratory of Functional and Molecular Imaging, National Institute of Neurological Disorders and Stroke, National Institutes of Health, Bethesda, MD, 20892, United States; c Translational Neuroradiology Section, National Institute of Neurological Disorders and Stroke, National Institutes of Health, Bethesda, MD, 20892, United States

**Keywords:** Multiple sclerosis (MS), Magnetic resonance imaging (MRI), MR Microscopy

## Abstract

**Purpose:**

To perform magnetic resonance microscopy (MRM) on human cortex and a cortical lesion as well as the adjacent normal appearing white matter. To shed light on the origins of MRI contrast by comparison with histochemical and immunostaining.

**Methods:**

3D MRM at a nominal isotropic resolution of 15 and 18 μm was performed on 2 blocks of tissue from the brain of a 77-year-old man who had MS for 47 years. One block contained normal appearing cortical gray matter (CN block) and adjacent normal appearing white matter (NAWM), and the other also included a cortical lesion (CL block). Postmortem ex-vivo MRI was performed at 11.7T using a custom solenoid coil and T_2_*-weighted 3D GRE sequence. Histochemical and immunostaining were done after paraffin embedding for iron, myelin, oligodendrocytes, neurons, blood vessels, macrophages and microglia, and astrocytes.

**Results:**

MRM could identify individual iron-laden oligodendrocytes with high sensitivity (70% decrease in signal compared to surrounding) in CN and CL blocks, as well as some iron-laden activated macrophages and microglia. Iron-deficient oligodendrocytes seemed to cause relative increase in MRI signal within the cortical lesion. High concentration of myelin in the white matter was primarily responsible for its hypointense appearance relative to the cortex, however, signal variations within NAWM could be attributed to changes in density of iron-laden oligodendrocytes.

**Conclusion:**

Changes in iron accumulation within cells gave rise to imaging contrast seen between cortical lesions and normal cortex, as well as the patchy signal in NAWM. Densely packed myelin and collagen deposition also contributed to MRM signal changes. Even though we studied only one block each from normal appearing and cortical lesions, such studies can help better understand the origins of histopathological and microstructural correlates of MRI signal changes in multiple sclerosis and contextualize the interpretation of lower-resolution in vivo MRI scans.

## 1. Introduction

MRI has long been used to infer microstructural changes seen in neurological diseases, such as multiple sclerosis (MS), with high sensitivity. Changes to T_1_, T_2_, and T_2_* relaxation times, magnetization transfer (MT), apparent diffusion coefficient and diffusion anisotropy of water, as well as susceptibility and phase imaging, all provide a window into the microenvironment of the tissue, and many of these are used extensively in clinic for patient management (for a recent review see Petracca et al.). (Petracca et al., 2018) However, signal from MRI sequences typically used in clinical settings are not intrinsically tied to biological processes, and it is sometimes difficult to draw inferences to the specific pathological changes that cause these changes. (Petiet et al., 2018)

MRI signal changes typically seen in the brain and spinal cord of patients with MS are often assumed to be a combination of gliosis, edema, loss of neurons and myelin, and axonal transection. (Filippi and Rocca, 2011) In addition, imbalance of metals (such as iron and zinc) associated with oligodendrocyte damage and inflammation in MS (Todorich et al., 2009; Hametner et al., 2013; Popescu et al., 2017) can lead to MRI signal changes. Some studies have shown a loss of iron from normal appearing white matter (WM), while others have reported an increase in iron concentration in the deep gray matter (GM). (Stankiewicz et al., 2007; Walton and Kaufmann,1984; Craelius et al., 1982; Stankiewicz et al., 2014; Drayer et al., 1987; Bakshi et al., 2002) Due to its high sensitivity to iron, in-vivo magnetic resonance imaging (MRI), especially using T_2_*-weighted sequences, is ideally suited to investigate the role of iron in pathophysiology. However, there is an enormous gap between in-vivo imaging (mm scale) and pathology (sub-μm scale), which needs to be bridged to better understand the pathological correlates of MRI signal changes.

We have previously reported postmortem imaging and the use of 3D-printed cutting boxes to accurately target small lesions seen on MRI for subsequent histopathological analysis. (Absinta et al., 2014; Luciano et al., 2016; Absinta et al., 2015) Postmortem whole hemisphere MRI at isotropic resolutions on the order of 150–450 μm has led to identification of MRI signal changes in GM and WM. However, such studies still do not offer clarity about the specific origin of MRI signal changes in affected tissue. MRI at resolutions of 20–50 μm begins to approach single-cell resolution and may help in this endeavor. (Badea and Johnson, 2013) MRI at these high resolutions is referred to as magnetic resonance microscopy (MRM). The purpose of this study was to perform high resolution MRM of human cortex, adjacent NAWM, and a cortical lesion in postmortem human tissue, with corresponding histopathology, to establish an MRM protocol relevant for MS, better understand the sources of MRI signal, and ultimately facilitate proper in vivo interpretation.

## 2. Methods

Right hemisphere and brainstem were obtained postmortem from a 77-year-old man with secondary progressive MS. The next of kin donated this tissue for scientific research at the NIH. The patient was formally diagnosed with MS 34 years before death, with initial symptoms of blurred vision and paresthesia dating back to 47 years before death. Over the years, the patient suffered multiple clinical relapses, which included generalized weakness with gait difficulties. He was lost to followup at the NIH for about 30 years prior to his death, and therefore in-vivo MRI scans were not available for this study. He suffered respiratory failure 4 months before death due to aspiration pneumonia and sepsis. Postmortem interval was 15 h.

### 2.1. Tissue preparation and ex-vivo MRI

The postmortem CNS tissue donated to the lab for scientific research was first fixed in 10% formalin for two weeks and then stored in 1% formalin to avoid over-fixation. High-resolution, whole hemisphere MRI was performed on the donated tissue to identify abnormal regions, as described elsewhere. (Absinta et al., 2014) Briefly, images were placed in a postmortem imaging container filled with Fomblin (Solvay Solexis, West Deptford, NJ), which closely fit into a 32-channel head coil (NOVA medical, Wilmington MA). Images were acquired on a Magnetom 7T MRI system (Siemens, Malvern, PA) equipped with a circular-polarized transmit/32-channel receive coil. Imaging included a T_2_*-weighted sequence (3D gradient-echo sequence, TR = 60 ms, multiple TEs of 6.09, 15.99, 25.89, 35.79 ms, FA = 10°, 4 averages, 88 slices, 0.42 mm isotropic resolution, acquisition time = 2.25 h per 3D slab).

### 2.2. Tissue dissection and magnetic resonance microscopy

A cutting box was 3D-printed and tissue cut into slabs using the method described in detail elsewhere. (Absinta et al., 2014; Luciano et al., 2016) The tissue slabs corresponding to two regions of interest were identified using the ex-vivo images and two smaller blocks of tissue, approximately 125 and 250 mm^3^ in size (approximate maximum size of 12 mm × 4.5 mm × 4.5 mm), were dissected for MRM. MRM was performed on an 11.7T MRI system (Avance III, Bruker Corporation, Billerica, MA) equipped with a 12-cm gradient coil (Resonance Research Inc, Billerica, MA) with maximum gradient strength of 100 G/cm. The targeted tissue blocks were moved to 6-mm glass test tubes (Fisher Scientific) filled with Fomblin and imaged using a custom-built solenoid transmit-receive coil of 6-mm inner diameter and 10-mm length, with a custom-built transmit/receive switch and preamplifier proximal to the coil. T_2_* -weighted images were acquired (3D gradient-echo sequence, TR = 100 ms, TE = 10 ms (minimize), FA = 30°, 15 and 18 μm isotropic resolution depending on size of block, acquisition time of 36 and 56 h) for the CN and CL block respectively.

### 2.3. Histopathology

After MRM, the tissue blocks were paraffin-embedded and sectioned at 5 μm. Histochemistry and immunohistochemistry were performed on selected serial slides, and included staining for non-heme iron (using 3,3'-Diaminobenzidine or *DAB-Turnbull*) (Meguro et al., 2003), myelin (using Luxol fast blue-periodic acid Schiff or *LFB-PAS*), (Shanklin and Nassar, 1959) oligodendrocytes (using antibodies to aspartoacylase or *ASPA*; Genetex), (Moffett et al., 2011) neurons (neuronal nuclear antigen or *NeuN*; Abcam), (Gusel’nikova and Korzhevskiy, 2015) phagocytic macrophages and microglia (CD68; Thermo-Fisher), (Kunisch et al., 2004) astrocytes (glial fibrillary acidic protein or *GFAP*; DAKO), (Reyaz et al., 2005) and blood vessels (CD31 antibody; Abcam). (Vecchi et al., 1994)

In order to accurately localize iron and MRI signal to specific cell types, double staining with GFAP, CD68, NeuN, and ASPA was processed for DAB-Turnbull. After iron staining, sections were rinsed with distilled water, and consecutive pre-treatments of antigen retrieval were carried out on all slides. After blocking with 2.5 N horse serum, primary antibody incubation was performed. For recognition of the primary antibodies, slides were then incubated 30 min with horse anti-rabbit IgG or horse anti-mouse IgG alkaline phosphatase secondary antibodies. Slides were then washed with TBST, and sections were developed with blue chromogen (Vector blue, Vector Lab), dehydrated, and cover-slipped. Stained sections were visualized using Zeiss Microscope, camera, and Zeiss Zen Blue software. To compare with MRM, histology images were acquired at 100x magnification and stitched together using Zeiss mechanized platform and software. MRM images were manually resliced to match the histological images for ease of comparison. Histological images were also acquired at 400x (sometimes 600x) magnification at selected locations for better visualization of colocalized staining as well as morphological identification of cell types.

### 2.4. Quantification

Signal intensity profile plots were obtained 10 randomly chosen punctate hypointensities from a 4x-magnified, intensity-normalized image of the normal cortex (using Medical Image Processing, Analysis, and Visualization tools or MIPAV, nitrc.org; and Matlab, Mathworks Inc, Natick, MA). Average and standard deviation of the noise was obtained from the background voxels and plotted along with the profile plots. Signal-to-noise ratio was calculated by dividing the average signal in the cortex to the standard deviation of the noise in the background. Contrast-to-noise between the punctate hypointensities and the surrounding cortex was calculated as the ratio of the difference in signal between microstructures to the standard deviation of the noise in the background.

Finally, percentage of oligodendrocytes showing positive and negative iron signal were calculated from normal cortex and lesion region. Signal intensity profile plots were also obtained from 5 randomly selected oligodendrocytes each in the normal cortex and lesional region of the ASPA and DAB-Turnbull double stained slide, after converting the images to monochrome (using functions in Matlab, The Mathworks, Natick, MA). The profile plots were aligned to the nadir signal in the profile plots due to size variations.

## 3. Results

**Fig. 1.**
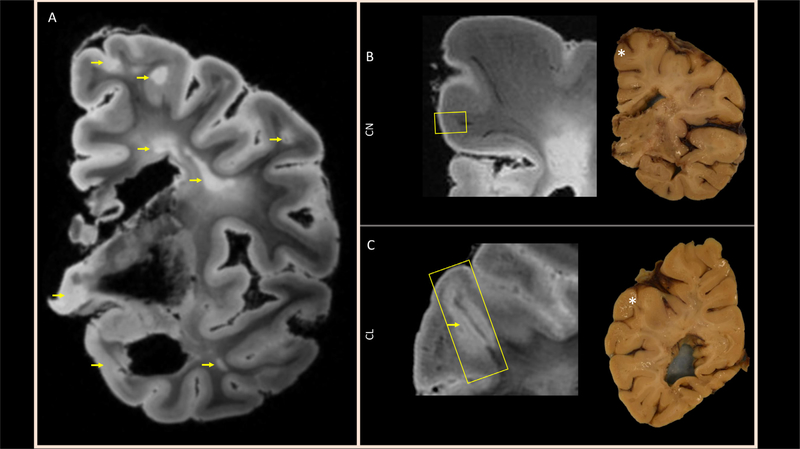
Preparation of the tissue for magnetic resonance microscopy (MRM): (A) Representative slice from T_2_* -weighted ex-vivo whole-hemisphere MRI depicts cortical, subcortical, and deep WM lesions (arrows). From images like these, small regions highlighted by the yellow boxes on zoomed-in ex-vivo MRI (left side of panels B and C) and localized with asterisks on photographs of the tissue slab surfaces (right side of panels B and C), encompassing (B) normal appearing cortex and WM (CN block) and (C) a leukocortical lesion (CL block), were excised for magnetic resonance microscopy (MRM). Note that the MRI image shown in (C) is from deep inside the 1-cm thick slab, but the cortical lesion is not visible on the slab surface.

Postmortem imaging of the right hemisphere revealed numerous cortical, subcortical, deep WM, and periventricular lesions spread throughout the brain, as seen often in patients with longstanding MS (Fig 1A). A cutting box was used for accurately sectioning the brain, and two small regions were selected for further study using MRM. The region of dissection is highlighted by yellow boxes on the zoomed-in ex-vivo MRI images on the left side of Fig 1B and 1C. Since the dissected region was located deeper within the tissue slab depicted on the right side of Fig 1B and 1C, an asterisk is used to denote the approximate surface location of the dissected region. The first block included normal appearing cortex and normal appearing white matter (CN, Fig 1B), and the other block also included a leukocortical lesion (CL, Fig 1C).

**Fig. 2.**
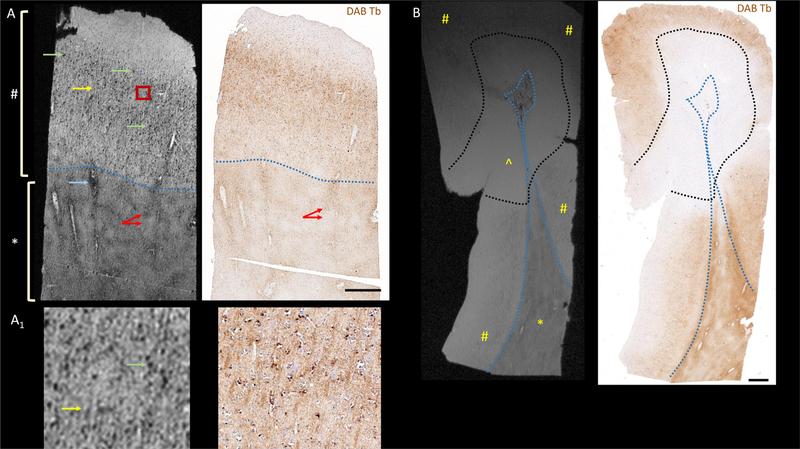
Imaging features and origins of the magnetic resonance microscopy (MRM) signal: MRM (left) and corresponding DAB-Turnbull staining (for iron in brown and hematoxylin counterstain in blue, right, 100x) are shown from (A) normal-appearing cortex/white matter and (B) a block containing leukocortical lesion. Normal appearing cortex (#), normal appearing WM (*), and cortical region of the leukocortical lesion (ˆ) are marked. Other features marked on (A) include punctate (green arrows) and linear (yellow arrows) hypointensities in the cortex, a region of patchy signal in the WM (red arrows), and a dark rim around a blood vessel (blue arrow). Most of these MRM features are also appreciated in DAB-Turnbull stain for iron, except for the overall difference between WM (lower signal in the MRI) and GM. Approximate boundary between GM and WM is demarcated with blue dotted line, and leukocortical lesion in (B) with black dotted line. Please refer to LFB-PAS staining of the CL block in Supplementary Figure 1 to see the extent of the demyelination. (A_1_) A representative portion of the cortical layer, approximately marked by a red box in (A), is magnified to show the punctate (green arrow) and linear (yellow arrow) hypointensities.

Fig. 2 compares the MRM images of both blocks with iron staining (DAB-Turnbull in brown, and hematoxylin counter stain in blue) performed on an identical location. Cortical (marked with #) and NAWM (marked with *) regions could be easily differentiated on the MRM (Fig 2 left panels in all subfigures, separated by blue dotted line for clarity in A and B) and on myelin staining (LFB-PAS, left panel of supplementary Fig 1A and B), but not on the iron-stained images (DAB-Turnbull, right panel of all subfigures in Fig 2).

MRM of the CN block (Fig 2A, left panel) revealed numerous punctate hypointensities (green arrow) in the middle and deeper layers of the normal appearing cortex. The superficial layers of the cortex were devoid of these punctate hypointensities. Lines of hypointense signal (yellow arrow) extended perpendicularly to the cortical surface throughout the deeper two-thirds of the cortical thickness. The pattern of the punctate hypointensities in the MRM visually corresponded with the location of iron as identified by the DAB-Turnbull staining (Fig 2A, right panel). These punctate and linear signals are better appreciated in the magnified images of cortical region of Fig 2A(Fig 2A_1_).

Similarly, in the MRM of the CL block (Fig 2B, left panel), cortical region of the leukocortical lesion (marked by ˆ, and demarcated by black dotted line) was easily identified as being hyperintense in comparison with the middle and deeper layers of normal cortex (marked by #). It should be noted that tissue rotation during embedding caused a mismatch between the slice angle during MRM and histopathology. We used simple multiplanar reformatting to match these angles (Supplementary Fig. 2A). This lesion is also identified as a region of myelin pallor in the LFB-PAS staining (Supplementary Fig 1B left panel, demarcated by black dotted line). Note that there is a signal roll-off in the MRM image of the CL block, due to B1-inhomogeniety as the CL block was larger than the sensitive portion of the RF coil used in imaging. Increasing the length of the solenoid coil, and changing the acquisition parameters such as field-of-view and number of points, could have improved the visibility of the lesion. Normal appearing cortex in the CL block appeared consistent with normal appearing cortex in the CN block, in terms of visibility of punctate and linear hypointensities, despite the fact that the CL block was imaged at a lower resolution due to its bigger size. DAB-Turnbull staining of the CL block (Fig 2B, right panel) clearly shows a stark loss of iron in the cortical region of the leukocortical lesion in comparison with normal appearing GM.

Another prominent feature that could be identified in the CN and CL blocks is patchy signal in the WM (red arrows in Fig 2A), which visually corresponded to the patchiness in iron signal in the same region. In addition, there were dark continuous rims seen on most of the larger blood vessels in both the cortex and WM (light blue arrow in Fig 2A, left panel). This signal did not visually correspond with either iron accumulation or spatial variations in myelin concentration seen on LFB-PAS stain, which was instead homogeneous in the WM (Supplementary Fig 1A, left panel). Sirius red (Supplementary Fig 1 A and B, right panels) staining showed dense deposition of collagen along the walls of some of the vessel, which could be a reason for hypointense MRM signal in this region.

**Fig. 3.**
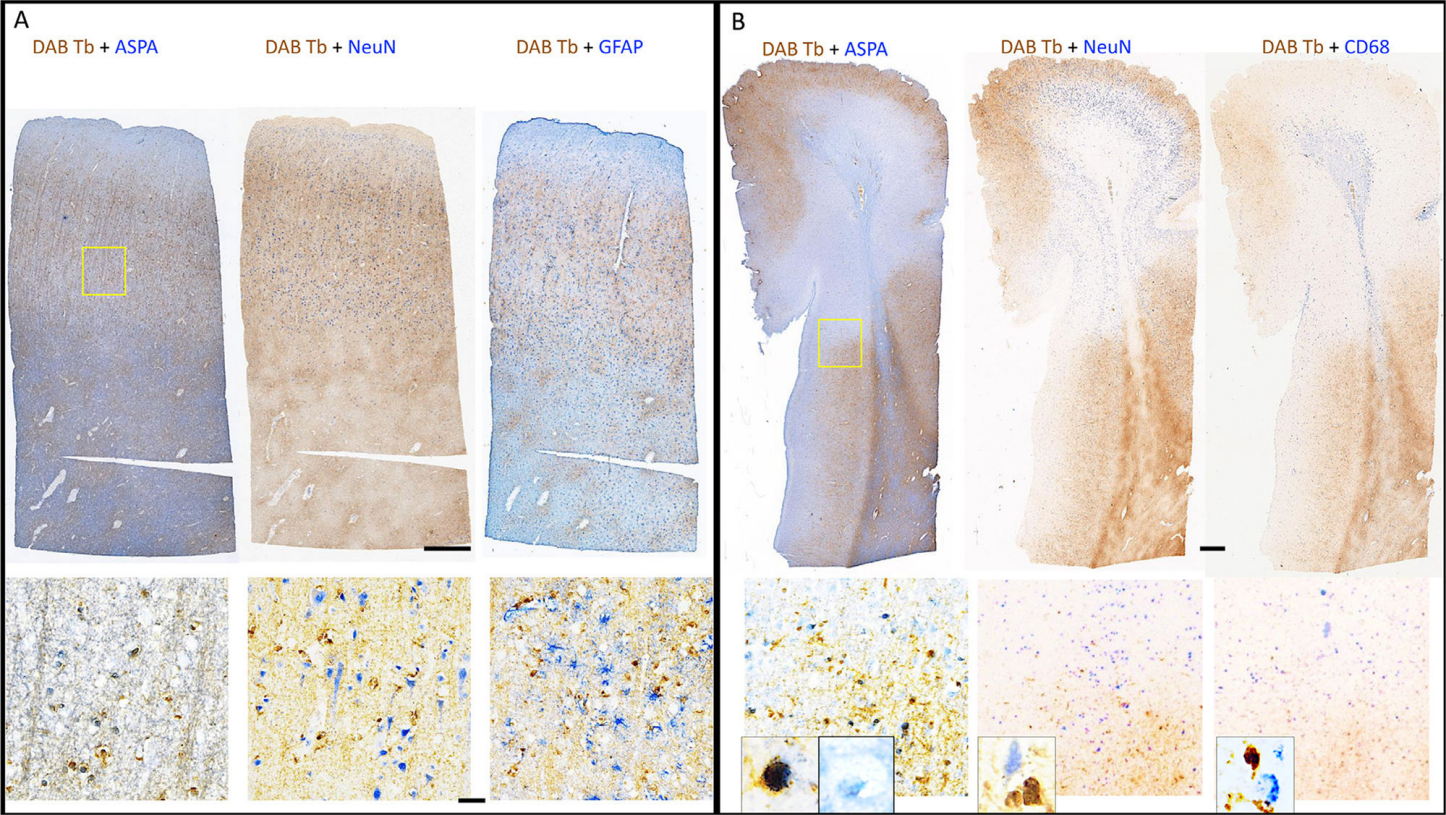
Identity of iron-positive cells: Double staining of (A) normal-appearing cortex/white matter and (B) a block containing leukocortical lesion stained with DAB-Turnbull (in brown) for iron and counter-stained in blue with antibodies to ASPA (left column), NeuN (middle column), GFAP (right column in A), and CD68 (right column in B) at 100x. In non-demyelinated tissue (A), iron primarily colocalizes with ASPA in both blocks, but at the edge of the leukocortical lesion there is some colocalization with CD68. Iron does not colocalize with NeuN or GFAP. Higher magnification image (400x) from region marked with yellow box (top right of A and B) is shown in bottom row, with further magnified image (600x) in inset of bottom row in (B). Scale bar is 1 mm for top row and 50 μm for bottom row.

In order to identify the cellular location of iron, a series of double stains for iron and various cell types were performed in both CN (Fig 3A) and CL (Fig 3B) blocks. Iron signal (in brown) was colocalized with oligodendrocyte cell bodies (left column in 3A and 3B); in addition, there is a background of brown signal that could correspond either to nonspecific staining or, more likely, to iron within myelin. Loss of cellular iron in oligodendrocytes was seen within the cortical portion of the leukocortical lesion (Fig 3B, left column). Specifically, the bottom left panel of Fig 3B shows colocalization of iron and ASPA in the lower half, as well as a background of iron likely contained within myelin, but only ASPA signal in the top half. Iron signal did not colocalize with neurons (middle column in 3A and 3B), or astrocytes (right column in Fig 3A), in either the CN or CL blocks. Rarely, some macrophages/microglia showed colocalization with iron signal inside the brain parenchyma (right column, Fig 3B). Magnified (400x) images (from regions in yellow box at the edge of the lesion encompassing some normal appearing cortex) are shown in the bottom row of Fig 3. The loss of iron can be appreciated in the lesional region on all the images. Images at higher magnification (600x, shown as insets in bottom row of Fig 3B) show colocalization of DAB-Turnbull and ASPA on the left within normal cortex, and positive ASPA signal without iron signal from within lesional region. Similar lack of colocalization can be appreciated in the 600x magnified images of DAB-Turnbull + NeuN as well as DAB-Turnbull + CD68.

The signal-to-noise ratio of the MRM was 23.7, and the contrast-to-noise ratio between the punctate hypointensities and the surrounding cortex was 17.6. Signal intensity profile plots were drawn on punctate hypointensities, as shown in Supplementary Fig. 2B (inset, red line). Average signal intensity profile plots from 10 such punctate hypointensities (blue line, Supplementary Fig. 2B) show a clear dip, with a calculated full-width-at-half-maximum of about 20 μm corresponding to about one voxel in the original MRM image. The signal within these hypointensities was 70% less than the surrounding cortex.

Closer look at two regions in the ASPA + DAB-Turnbull double stained slide revealed that 84 of the 94 (89%) oligodendrocytes in the normal cortex contained iron, whereas this was true only for only 8 of 57 (14%) oligodendrocytes in the lesional region (Supplementary Fig. 2C). Profile plots drawn across 5 randomly selected oligodendrocytes with iron signal, and 5 without iron signal, showed significant signal intensity differences between the two (Supplementary Fig. 2D). The plot reveals significant differences in signal intensity in the immunohistochemistry images, which enabled easy differentiation of oligodendrocytes with and without iron.

## 4. Discussion

T_2_*-weighted MRM sequence at 15–20 μm isotropic resolution was able to identify individual iron-laden oligodendrocytes and, more rarely, macrophages/microglia in postmortem fixed tissue from an MS patient. Loss of iron within the cortical regions of the leukocortical lesion caused it to have an overall brighter signal compared to normal cortex. In addition, variable density of iron-laden oligodendrocytes caused patchy MRM signal changes within NAWM (not shown in detail). The boundary between cortex and WM was not distinguishable in the iron stains, indicating that the high density of myelin itself, rather than iron within myelin processes in NAWM, was primarily responsible for hypointense MRM signal in NAWM. Finally, dark rims could be seen around many blood vessels in the cortex and NAWM, which seemed to arise from collagen deposition during vascular remodeling in tissue.

Use of high magnetic fields with high gradient strengths and custom-built RF coils, and averaging the MR signal over many hours, made it possible to image single cells and processes in their native environment, albeit in fixed tissue. MRM has been performed using intrinsic contrast at high fields and specialized hardware at even higher resolutions (as high as 5 μm (Filippi and Rocca, 2011)), enabling visualization of individual isolated cells, such as neurons, and identification of subcellular structures, such as nuclei. (Aguayo et al., 1986; Schoeniger et al., 1994; Bowtell et al., 1995; Lee et al., 2015; Lee et al., 2017) Other studies have looked at the microenvironment of cells in tissue such as rat spinal cord. (Flint et al., 2009) The MRM contrast reported herein originated largely from differences in concentration of iron, myelin, and collagen. Iron and myelin are both known to be strong contributors to contrast on T_2_*-weighted images. (Duyn, 2011) Protons associated with macro-molecules such as collagen (deposited on blood vessels), and starch-rich cells (corpora amylacea, not explored herein) would have very short T_2_* relativity, and would appear hypointense.

NAWM appeared relatively dark on MRM when compared to the cortex and WM lesion on the MRM which could be attributed directly to the high density of myelin in the white matter. This is in line with previous studies that have reported a direct correlation between T2* signal intensity and myelin content on a larger scale of 2 × 2 × 2 mm^3^. (Li et al., 2009) However, subtle variations in the concentration of myelin, if any, could not be identified on LFB-PAS. This suggests that the MRM pulse sequence used herein was sensitive only to large changes in myelin density. On the other hand, patchy signal variations in the WM, over an estimated hundreds of microns across, seemed to be directly related to changes in density of iron-laden cells. This may be explained by the fact that iron is stored in super-paramagnetic form H-ferritin within oligodendrocytes with susceptibility of the order of +500 ppm. (Schenck,1996) By contrast, myelin has anisotropic susceptibility ranging between ± 10 ppm. Even so, the significant loss of GM-WM T_2_/T_2_* contrast with demyelination has been quantified in animal models, (Nair et al., 2005; J. Lee et al., 2012) which has been attributed to the very large changes to the density of myelin in the WM. Given these large susceptibility differences, it is conceivable that imaging such as that described herein is possible even at lower field strengths. However, a high sensitivity RF coil with optimal fill factor for the given tissue, coupled with high performance gradient coil, would be required. Furthermore, TR, TE, and flip angle, as well as signal averaging, would have to be adjusted for changes to relaxivity and loss of SNR at lower fields.

Iron is known to play an important role in the disease process in MS. It is essential in metabolic processes as its oxidative state is easily changed. Excess iron accumulation has been postulated to relate to neurodegeneration, as in high concentration iron may contribute to disease via formation of reactive oxygen species, (Dixon and Stockwell, 2014; Gammella et al., 2016) while iron deficiency can lead to demyelination and reduced myelination in development. (D.L. Lee et al., 2012) Although neurons are known to accumulate iron, especially with age, glia are thought to have an essential role in iron homeostasis in the brain. (Mills et al., 2010) Chronic inflammation associated with MS targets myelin-making oligodendrocytes, (Lucchinetti et al., 2000) which are known (and shown herein) to have high iron levels in both their cell bodies and processes. (Benkovic and Connor, 1993) It is therefore reasonable that iron can play an important role in identifying pathophysiological changes in neurological diseases, especially at high field strengths.

Iron-rich oligodendrocytes are known to be present alongside neurons in cortical GM. (Connor and Menzies, 1996) Cortical regions of the leukocortical lesion studied herein were iron-deficient, however, making it appear brighter than its surroundings on the MRM. T_2_* hyperintensity in the cortex has previously been associated with cortical lesions, even on in-vivo MRI. (D. Kidd et al., 1999;D. Pitt et al., 2010) The identification of iron-poor oligodendrocytes at the edge of the leukocortical lesion (Fig 3B) has uncertain significance, but it raises the possibility that iron release from oligodendrocytes may be relevant to the pathophysiology of demyelination, e.g. by altering the metabolism of these cells. Alternatively, it is possible that the iron-deficient oligodendrocytes are newly differentiated cells attempting to repair inflammatory injury at the site. (Prineas et al., 1989) Lack of many iron-laden macrophages in the brain parenchyma may be attributed to the longstanding MS in this subject, and probably lack of active demyelinating lesions. However, a more detailed study of people who are closely followed during their disease progression would be needed to answer these questions.

MRM revealed a hypointense rim surrounding most of the blood vessels in the cortex and WM in addition to patchy signal (Fig 2). Commensurate with this, a rim of collagen deposition is seen around some of the larger blood vessels in Sirius red staining, but not in the iron staining. This observation adds evidence that the dark signal is seen around blood vessels could be a result of water exclusion from collagen deposition, (Absinta et al., 2019) short T_2_* relaxation times of macro-molecules. Vascular hypointensity is commonly seen within MS lesions in the lower-resolution in-vivo scans, and is referred to as the “central vein sign.” (Lummel et al., 2011; Sati et al., 2016) It is possible that more subtle vascular remodeling outside lesions are visible in the high-resolution, highly sensitive MRM scans reported herein. Nevertheless, there were no hypointense rims visible within either the cortical or the white matter portions of the leukocortical lesion.

While utmost care was used to depict correspondence between MRM and histopathological images, nonlinear distortions and tearing during histological processing, and also differences in cutting location from one slide to the next precluded automated methods for MRI-histology registration. Since the tissue in question was relatively small, and histology was done after MRM, it was relatively easy to perform the match manually, the success of which can be appreciated when looking at some of the larger features such as blood vessels on the MRI and histopathology. Automated registration algorithms also introduce smoothing to the images, which could be detrimental to visualizing punctate hypointensities. Therefore, quantitative correlation analyses was not performed on MRM and histopathological images, and we resorted to a qualitative visual comparison for the study. In addition, a major limitation of this study was the limited number of samples studied herein, in that all samples were obtained postmortem from a single subject. Further MRM studies are planned to confirm and expand these findings, including MRM of other brain structures.

With respect to the translation of the findings from this study to the interpretation of in vivo MRI, it is important to point out that hyperintensities in T_2_*-weighted imaging as well as hypointensities in T_1_-weighted images of cortex have been observed in-vivo, and are associated with disability in MS. (D. Kidd et al., 1999; D. Pitt et al., 2010; Beck et al., 2018) In addition, postmortem MRI has revealed hyperintense cortical lesions confirmed by histopathology. (D. Kidd et al., 1999; Kilsdonk et al., 2016) However, the proportion of cortical lesions detectable on MRI remains a small fraction of that typically identified in postmortem histopathology, probably due to issues with MRI sensitivity and resolution. It is seldom possible to image highly disabled patients directly before death for a reliable comparison. Postmortem imaging and MRM studies coupled with corresponding histopathology can provide a vital link to better understand the histopathological correlates of MRI signal changes, thereby providing an opportunity to improve in-vivo acquisition. For example, based on the results of this study, we have developed a high-resolution T2*-weighted sequence with CSF-suppression for better visualization of cortical lesions. Such a sequence has now been validated in MS and healthy volunteers at 3T in our lab, and comparison with other sequences for imaging cortical lesions in MS on the 3T and 7T clinical scanners performed. (Beck et al., 2020)

In summary, our study sheds light into pathophysiological changes that can cause distinct MR signal changes and helps contextualize signal changes seen *in-vivo* MRI. It is clear that oligodendrocytes contain enough iron to allow detection of single cells at high resolutions in the native environment. In addition to iron, various imaging features could also be attributed to myelin and collagen deposition. The heterogeneity of changes seen at these very high resolutions indicates that the context of in-vivo MRI contrast changes must be considered carefully. Better understanding of this relationship can help us design pulse sequences to improve the specificity of the in-vivo MRI.

## Supplementary Material

1
