# Supplementary material for: Ex vivo MR microscopy of a human brain with multiple sclerosis: Visualizing individual cells in tissue using intrinsic iron
Source: Neuroimage. Author manuscript; Available in PMC 2021 Jan 17. (PMC7811778; doi:10.1016/j.neuroimage.2020.117285)
Supplement: 1 [file NIHMS1659544-supplement-1.docx]

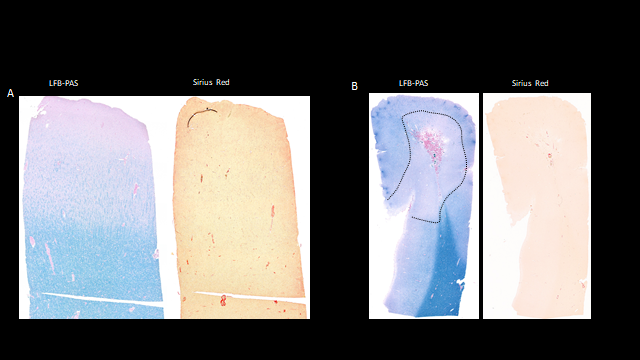


**Supplementary Figure 1:** **Myelin and collagen stains explain other signal changes in MRM:** Luxol fast blue–periodic acid Schiff staining (left panel) of (A) normal-appearing cortex/white matter and (B) a block including leukocortical lesion (at 100x) clearly depicts the GM/WM boundary and the region of leukocortical lesion (marked with ^ in B, demarcated with black dotted line). Sirius red (right panel) stains the boundary of the larger vessels dark, indicating the presence of collagen in both blocks.

**Supplementary Figure 2: A. Alignment between ex-vivo MRI and MR Microscopy of the CL block: Ex-vivo** MRI depicted in Figure 1C (left), MRM performed in the same orientation as Figure 1C (middle), and reoriented MRM to match with histology as shown in Figure 2B (right) for the CL block.  **B.** **Signal intensity profile plot across punctate hypointensities on the MRM:** Average and standard deviation of MRM signal intensities, normalized to maximum signal in image, across 10 randomly selected punctate hypointensities in the mid-cortex region (after 4x magnification, representative regions shown with red lines in inset image on left). Noise metric (mean + 2*standard deviation, green line) from 3700 voxels in the background (approximate region shown in green box in inset image on right) is also plotted as a constant line for reference. **C.** **Percentage of iron-positive oligodendrocytes within lesion and in normal cortex:** Iron signal could be manually identified in only 14% of the oligodendrocytes within the lesion (red box on inset image), compared to 90% of the oligodendrocytes in normal cortex (yellow box on inset image) region in the ASPA-DAB-Turnbull double stained slides from Fig 3B. **D.** **Signal intensity profile plot across oligodendrocytes within lesion and in normal cortex:** Average and standard deviation of image intensities across randomly selected oligodendrocytes, 5 of which showed colocalization with iron (brown line, left inset), and 5 of which had no iron signal (blue line, right inset) from the ASPA and DAB-Turnbull double-stained slide pictured in 3B. These plots show that the background and iron-positive oligodendrocytes could be easily differentiated from iron-negative oligodendrocytes.
